# Development and characterization of 17 microsatellite markers for *Sonchus oleraceus*

**DOI:** 10.1002/aps3.11329

**Published:** 2020-02-24

**Authors:** Seon‐Hee Kim, Hye‐Been Kim, Min‐Seong Cho, Chang‐Seok Kim, Seung‐Chul Kim

**Affiliations:** ^1^ Department of Biological Sciences Sungkyunkwan University 2066 Seobu‐ro Suwon 16419 Republic of Korea; ^2^ Highland Agriculture Research Institute National Institute of Agricultural Sciences Rural Development Administration (RDA) Gangwon‐do 25342 Republic of Korea

**Keywords:** Asteraceae, invasive species, microsatellite markers, *Sonchus oleraceus*, weeds

## Abstract

**Premise:**

The common sowthistle, *Sonchus oleraceus* (Asteraceae), is a globally invasive weedy species. In order to investigate its genetic diversity, population genetic structure, and evolutionary history, we developed and characterized nuclear simple sequence repeat markers (SSRs or microsatellites).

**Methods and Results:**

Seventeen microsatellite primer pairs were developed based on the Illumina sequence data. Ten developed SSR loci were polymorphic in four populations sampled from broad geographical regions. The number of alleles per locus ranged from one to 11, and the levels of observed and expected heterozygosity ranged from 0.000 to 1.000 and from 0.000 to 0.801, respectively. Up to 82% of the newly developed primer pairs were successfully amplified in the congeneric taxa *S. asper*,* S. asper* subsp. *glaucescens*,* S. canariensis*, and *S. palmensis*.

**Conclusions:**

The SSR markers developed in this study will be useful for future population genetic studies on *S. oleraceus* and other congeneric species.

The common sowthistle, *Sonchus oleraceus* L. (Asteraceae), is an annual or biennial herb species. As one of the cosmopolitan weed species in the genus *Sonchus* L., it is found in almost every ice‐free part of the world (Funk et al., 2009). *Sonchus oleraceus* and *S. asper* (L.) Hill have great allelopathic potential and are competitive even in weedy communities, making these species particularly troublesome weeds across grain‐growing regions. In addition, the evolution of herbicide resistance found in populations of *S. oleraceus* threatens the efficiency of chemical control methods (Cook et al., 2014). Although *S. oleraceus* is recognized as an invasive weed species, it also has various culinary uses and medicinal (antibacterial, anti‐inflammatory, and antioxidant) properties (Jimoh et al., 2011).

Seashore habitats in the Mediterranean region have been proposed to be the geographical origin of the common sowthistle (Boulos, 1960), although this is not confirmed. Stebbins et al. (1953) hypothesized that *S. oleraceus* (2*n* = 32) has an amphidiploid origin, involving hybridization between *S. asper* (2*n* = 18) and *S. tenerrimus* L. (2*n* = 14). Although chromosome numbers, morphological characteristics, and phylogenetic analysis provide some evidence to support this hypothesis, no corroborating results or conclusive evidence have been generated yet (Mejías and Andrés, 2004; Mejías et al., 2018). Therefore, in order to infer its phylogenetic divergence and expansion history on a local and global scale, it is of great importance to determine the origin of amphidiploid *S. oleraceus*.

Given their high level of polymorphism, co‐dominant inheritance patterns, and wide genomic distribution, microsatellites have been proven to be useful in estimating genetic diversity and characterizing population genetic structure, as well as for hybrid identification (Chen et al., 2017; Li et al., 2019). However, to our knowledge, no microsatellite markers have been developed for *S. oleraceus* or its closely related species despite their importance as agricultural weeds. In this study, we developed 10 polymorphic and seven monomorphic nuclear simple sequence repeat (SSR) markers for *S. oleraceus* and estimated the genetic diversity of four natural populations originating from broad geographical regions. In addition, we tested their applicability in four congeneric taxa (*S. asper*,* S. asper* subsp. *glaucescens* (Jord.) Ball, *S. canariensis* (Sch. Bip.) Boulos, and *S. palmensis* (Webb) Boulos). Our results suggest that the developed SSR markers will be valuable for future population genetic studies of *Sonchus* species.

## Methods And Results

Two individuals of *S. oleraceus* (voucher specimens: SKK Cho et al. SonO‐Uds2 from Korea and SEV 283322 from Spain; Appendix 1) were used to develop the microsatellite markers, and 72 individuals belonging to four populations from broad geographical regions (Korea, China, Germany, and Australia) were sampled in order to evaluate the polymorphisms of the target loci. Moreover, we used five individuals for each of four taxa of the genus *Sonchus* for cross‐amplification: *S. asper* subsp. *asper*,* S. asper* subsp. *glaucescens*,* S. canariensis*, and *S. palmensis* (Appendix 1). *Sonchus asper* (subg. *Sonchus* L.) represents a purported parental species in the hybrid origin of *S. oleraceus*, whereas *S. canariensis* and *S. palmensis* (subg. *Dendrosonchus* Sch. Bip. ex Boulos) represent two woody perennials endemic to the Canary Islands, Spain; these latter two species were selected to test the broader applicability of the developed microsatellite markers.

Total genomic DNA was extracted from silica gel–dried leaf tissues using the DNeasy Plant Mini Kit (QIAGEN, Carlsbad, California, USA), following the manufacturer instructions. An Illumina paired‐end genomic library was constructed using a TruSeq DNA LT Sample Prep Kit (Illumina, San Diego, California, USA), and the library sequencing was conducted with an Illumina HiSeq 4000 platform at the Macrogen Corporation (Seoul, Korea). All raw reads were submitted to the National Center for Biotechnology Information (NCBI) Sequence Read Archive (BioProject ID PRJNA577793). Altogether, 15.6 Gbp was sequenced and a total of 50,229,522 and 53,108,408 paired‐end reads were obtained for samples from Korea and Spain, respectively. Raw data were cleaned up by trimming the adapters and low‐quality reads with a custom script by the Macrogen Corporation, and the reads were assembled de novo using Velvet version 1.2.10 with default settings (Zerbino and Birney, 2008).

Microsatellite motifs with a repeat unit ranging from two to six nucleotides and a minimum number of six repeats were detected using MISA version 1.0.0 (Thiel et al., 2003) with the default parameters. A total of 685 regions were found. Primer pairs were designed using Primer3 version 2.3.6 (Koressaar and Remm, 2007) with the following settings: PCR product size of 100–300 bp, length from 12 to 48 bp, annealing temperature of 57–63°C, and GC content of 40–60%. The PCR amplifications were performed in a total volume of 20 μL, containing 1 μL of genomic template DNA, 0.5 μL of 10 mM forward primer with fluorescent dye (Table 1), 0.5 μL of 10 mM reverse primer, 2.5 μL (10× with 2.5 mM MgCl_2_) of PCR buffer, 0.5 μL of dNTPs of 10 mM, and 0.1 μL (1.25 U/μL) of *Taq* DNA polymerase (Inclone Biotech, Gyeonggi‐do, Korea). The PCR cycles were as follows: initial denaturation at 95°C for 1 min; 35 cycles of denaturation at 95°C for 20 s, annealing at 54–64°C for 40 s, and extension at 72°C for 60 s; and a final extension at 72°C for 5 min. The PCR products were detected electrophoretically on a 3% agarose gel.

**Table 1:** Characteristics of 17 microsatellite markers developed from *Sonchus oleraceus* genomic sequences.

Locus	Primer sequences (5′–3′)	Repeat motif	Allele size range (bp)	*A*	*T*_a_ (°C)	Fluorescent dye	GenBank accession no.
SO_06^a^	F: GTAGACTTGCCTCGCTCTCG	(TCTGCC)_4_	270, 276	2	54^c^	FAM	MN567078
	R: TGATCACTGGGAGACGCTTG						
SO_07	F: CTCCTTAGGTCGGCAAGTCA	(GAG)_5_	276–312	5	56^c^	HEX	MN567093
	R: CAATCCCCCACCAGTGACTC						
SO_10	F: CGCCGTCGTCAACTTTTTCA	(AGA)_4_	223–247	6	60	FAM	MN567094
	R: AGCCTCAAACCTTGCACTGT						
SO_2289^b^	F: AGGCACACACAGTTCTAGCA	(TGA)_4_	227	1	60	FAM	MN567086
	R: CTGACAGTTGACCAGGGTGT						
SO_2909^a^	F: CTTCTTGTGCCCCTTCTCGT	(AGT)_6_	251, 254	2	60	FAM	MN567087
	R: GGAAGCTAGAGAACGCCTCC						
SO_3270^a^	F: GAAGAAGGGCGGAGAGACTG	(GAA)_4_	169, 172	2	60	FAM	MN567088
	R: TGACTCCACCTCAACGTTGG						
SO_5917^a^	F: TCCACCACCCAAGAAGAAGC	(AGA)_4_	242, 245	2	60	FAM	MN567089
	R: GGGATTGCGAAGGTGGAGAA						
SO_6029	F: CGAACCCTACAATCTAGCATGC	(AAT)_4_	220, 229	2	60	FAM	MN567090
	R: TCTGCGGCTCGAGAAAAAGT						
SO_6786	F: CAGGGTGATATAATATGTGGTTGTG	(TA)_5_	280, 282	2	60	HEX	MN567091
	R: CACATCTCCTAAAACACCTTTTGGA						
SO_7064	F: CAACTCTGTATCGAGTGGGCA	(TC)_12_	260–278	8	60	HEX	MN567092
	R: AACTCCACCAACCTGCACAA						
SO_10093^a^	F: TCGCAACAAAACGGTTAGCA	(AT)_5_	278, 280	2	60	HEX	MN567079
	R: GGTCGAGGAGAGTGTACGTC						
SO_11084	F: GCTGATTCAGGTGGCCCATA	(AC)_8_	252–258	4	60	HEX	MN567080
	R: ATAATGCTGCAGGGGACACC						
SO_11221	F: AGTCGTTGCTTCCTTCCTTCT	(CA)_9_	270–276	4	60	HEX	MN567081
	R: TGTCAAAATACACCCCTAGAACTCA						
SO_11810	F: CCAAGTATGGTCCCACACCC	(CCATCT)_4_	185–215	6	60	HEX	MN567082
	R: CCACACGGAGGACAGTTCAA						
SO_12829	F: AGCTTTGTCTCTCACAGGCT	(CT)_9_	271–301	11	64	HEX	MN567083
	R: AAGATTGAGTTTGGCGGGGT						
SO_13164	F: CCCTCTCCCACGATCGATTG	(TCT)_4_	243, 270	2	60	HEX	MN567084
	R: TGAGGTGGAAGATGTGCGTG						
SO_20586^a^	F: GGTTCATTCCTGGCTTCCGA	(TCA)_4_	192, 198	2	60	FAM	MN567085
	R: TACGGACAAACTCACACCGG						

Note

*A* = number of alleles; *T*_a_ = annealing temperature.

a Fixed heterozygous locus.

b Monomorphic locus.

c Although most of the samples were amplified based on a higher *T*_a_ setting, some samples failed to amplify, and thus, lower annealing temperatures were used for PCR.

Of the 685 regions identified, 107 randomly selected primer pairs were tested for amplification efficiency by PCR using eight representative individuals of *S. oleraceus* sampled from four populations. Of these 107 primer pairs, 33 failed to amplify and the remaining 74 displayed clear bands and were suitable for further analysis. The PCR amplicons were labeled with fluorescent dye (HEX and FAM) and separated with a GeneScan 400 LIZ Size Standard (Applied Biosystems, Foster City, California, USA) at the Macrogen Corporation using an ABI 3730XL DNA Sequencer (Applied Biosystems). The microsatellite marker profiles were scored using GeneMapper version 5.0 (Applied Biosystems). Of the 74 successfully amplified loci, 10 were found to be polymorphic and seven were monomorphic (Table 1). The remaining 57 loci were excluded due to difficulty in obtaining clean peaks and unsuccessful amplification after labeling the forward primer with fluorescent dye. Population genetic parameters, including the number of alleles, expected heterozygosity, and observed heterozygosity, were calculated using GenAlEx version 6.503 (Peakall and Smouse, 2012). Significant deviations from Hardy–Weinberg equilibrium and linkage disequilibrium were estimated using GENEPOP 4.2 (Rousset, 2008) using the default parameters. The conventional chi‐square goodness‐of‐fit tests were performed to compare observed and expected genotypic frequencies (α = 0.05). Polymorphism information content, a value that is indicative of a measure of the informativeness of a genetic marker for linkage studies, was estimated using CERVUS version 3.0.7 (Kalinowski et al., 2007).

In four investigated populations of *S. oleraceus*, the number of alleles ranged from one to 11 (Table 1), polymorphism information content values ranged from 0.000 to 0.778, and levels of observed and expected heterozygosity ranged from 0.000 to 1.000 and from 0.000 to 0.801, respectively (Table 2). Significant deviation from Hardy–Weinberg equilibrium was detected in the four populations: Australia (eight loci), Germany (three loci), China (two loci), and Korea (one locus) (Table 2).

**Table 2:** Genetic properties of 17 newly developed microsatellite markers in four populations of *Sonchus oleraceus* in Eurasia and Australia.^a^

Locus	Australia (*N* = 10)				Germany (*N* = 20)				China (*N* = 22)				Korea (*N* = 20)			
	*A*	*H*_o_	*H*_e_	PIC	*A*	*H*_o_	*H*_e_	PIC	*A*	*H*_o_	*H*_e_	PIC	*A*	*H*_o_	*H*_e_	PIC
SO_06	2	1.000*	0.500	0.375	2	1.000	0.500	0.375	2	1.000	0.500	0.375	2	1.000	0.500	0.375
SO_07	2	1.000*	0.500	0.375	3	0.950*	0.596	0.521	3	0.682	0.563	0.487	3	1.000	0.614	0.539
SO_10	2	1.000	0.500	0.375	3	1.000	0.524	0.410	4	1.000	0.600	0.518	3	1.000	0.594	0.511
SO_2289	1	0.000	0.000	0.000	1	0.000	0.000	0.000	1	0.000	0.000	0.000	1	0.000	0.000	0.000
SO_2909	2	1.000*	0.500	0.375	2	1.000	0.500	0.375	2	1.000	0.500	0.375	2	1.000	0.500	0.375
SO_3270	2	1.000*	0.500	0.375	2	1.000	0.500	0.375	2	1.000	0.500	0.375	2	1.000	0.500	0.375
SO_5917	2	1.000*	0.500	0.375	2	1.000	0.500	0.375	2	1.000	0.500	0.375	2	1.000	0.500	0.375
SO_6029	2	0.400	0.320	0.269	2	0.950*	0.499	0.374	2	0.318	0.268	0.232	2	0.650	0.439	0.342
SO_6786	2	1.000*	0.500	0.375	1	0.000	0.000	0.000	2	0.000	0.165*	0.152	1	0.000	0.000	0.000
SO_7064	6	1.000	0.690	0.643	4	1.000	0.616	0.547	4	1.000	0.563	0.467	5	1.000	0.775	0.740
SO_10093	2	1.000*	0.500	0.375	2	1.000	0.500	0.375	2	1.000	0.500	0.375	2	1.000	0.500	0.375
SO_11084	1	0.000	0.000	0.000	4	1.000	0.606	0.528	1	0.000	0.000	0.000	3	1.000	0.594	0.511
SO_11221	3	1.000	0.640	0.563	3	1.000	0.594	0.511	3	0.909*	0.515	0.404	3	1.000	0.564	0.469
SO_11810	4	0.300	0.475	0.440	5	1.000	0.650	0.590	2	1.000	0.500	0.375	3	0.250	0.406	0.371
SO_12829	6	1.000	0.750	0.711	8	1.000*	0.801	0.778	3	1.000	0.522	0.407	4	1.000*	0.688	0.630
SO_13164	1	0.000	0.000	0.000	1	0.000	0.000	0.000	2	1.000	0.500	0.375	1	0.000	0.000	0.000
SO_20586	2	1.000*	0.500	0.375	2	1.000	0.500	0.375	2	1.000	0.500	0.375	2	1.000	0.500	0.375

Note

*A* = number of alleles per locus in each population; *H*_e_ = expected heterozygosity; *H*_o_ = observed heterozygosity; *N* = number of individuals sampled; PIC = polymorphism information content.

a Locality and voucher information are provided in Appendix 1.

* Locus showed significant deviation from Hardy–Weinberg equilibrium (*P* < 0.01) according to the chi‐square test.

The results of cross‐amplification in four congeneric taxa are shown Table 3. Out of 17 successfully amplified loci, six and seven loci were found to be polymorphic in *S. asper* and *S. asper* subsp. *glaucescens*, respectively. Furthermore, out of 14 successfully amplified loci in two woody *Sonchus* species (*S. canariensis* and *S. palmensis*), seven and five loci were polymorphic, respectively (Table 3). The high level of cross‐species transferability (>82%) suggests that the genus *Sonchus* has highly conserved sequences and that its species have recently diverged.

**Table 3:** Cross‐amplification of 17 microsatellite loci developed for *Sonchus oleraceus* in four related congeneric species.^a^

Locus	*S. asper* subsp. *glaucescens* (*N* = 5)		*S. asper* (*N* = 5)		*S. canariensis* (*N* = 5)		*S. palmensis* (*N* = 5)	
	*A*	Allele size range (bp)	*A*	Allele size range (bp)	*A*	Allele size range (bp)	*A*	Allele size range (bp)
SO_06	2	270, 276	2	270, 276	2	270, 276	2	270, 276
SO_07	2	291, 318	2	291, 318	1	276	2	276, 282
SO_10	4	238–247	1	247	3	232–247	2	232, 247
SO_2289	1	227	1	227	1	227	1	227
SO_2909	2	251, 254	2	251, 254	2	251, 254	2	251, 254
SO_3270	2	169, 172	2	169, 172	3	154–172	2	169, 172
SO_5917	2	242, 245	2	242, 245	2	242, 245	2	242, 245
SO_6029	3	220–232	2	220, 229	0	—	0	—
SO_6786	1	282	1	282	1	282	1	282
SO_7064	5	268–280	5	266–278	6	268–302	6	262–278
SO_10093	2	278, 280	2	280, 282	4	276–282	3	274–280
SO_11084	1	252	1	256	0	—	0	—
SO_11221	2	274, 276	4	270–274	3	272–276	2	272, 276
SO_11810	5	185–215	2	185, 197	2	197, 209	3	197–215
SO_12829	6	273–295	2	271, 287	0	—	0	—
SO_13164	2	243, 270	1	270	1	270	1	270
SO_20586	2	192, 198	2	192, 198	2	192, 198	2	192, 198

Note

— = unsuccessful amplification; *A* = number of alleles; *N* = number of individuals sampled.

a Locality and voucher information are provided in Appendix 1.

## Conclusions

In this study, we developed the first set of microsatellite markers for the genus *Sonchus*. Ten polymorphic markers are suitable for evaluating the population genetics and evolutionary history of *S. oleraceus*. These newly developed microsatellite markers displayed a high rate of cross‐amplification in congeneric species, demonstrating their applicability within the genus *Sonchus*.

## Author Contributions

S.‐H.K., C.‐S.K., and S.‐C.K. conceived and designed the project and collected the samples. C.‐S.K. obtained the funds from the Rural Development Administration. S.‐H.K., H.‐B.K., and M.‐S.C. performed the experiments. S.‐H.K. analyzed the data and wrote the draft of the manuscript. S.‐C.K. supervised the study and revised the manuscript. All authors approved the final version of the manuscript.

## Data Accessibility

All sequence information was deposited in the National Center for Biotechnology Information Sequence Read Archive (BioProject ID PRJNA577793). GenBank accession numbers are provided in Table 1.

## Appendix 1 Locality and voucher information for samples of *Sonchus oleraceus* and related species used in this study.

**Table:**

Species	Collection locality	Geographic coordinates	*N*	Voucher specimen accession no.^a^
*Sonchus oleraceus* L.	Jinjiangshan Park, Liaoning Sheng, China	40°07′58.9″N, 124°22′49.7″E	22	SKK060307 SKK060308
	Garmisch‐Partenkirchen, Germany	47°29′43.6″N, 11°06′30.8″E	20	SKK059414 SKK059420
	Singu‐ri, Jeollanam‐do, Korea	34°20′56.9″N, 127°02′15.5″E	20	SKK060323
	Bridgewater, Tasmania (TAS), Australia	42°44′15.9″S, 147°13′33.7″E	10	—
	Huelva, Los Marines, Spain	37°54′46″N, 006°37′07″W	1	SEV 283322
	Dokdo‐ri, Gyeongsangbuk‐do, Korea	37°14′29.7″N, 131°51′52.2″E	1	SKK Cho et al. SonO‐Uds2
*Sonchus asper* (L.) Hill	Singu‐ri, Jeollanam‐do, Korea	34°20′56.9″N, 127°02′15.5″E	5	SKK060322
*Sonchus asper* subsp. *glaucescens* (Jord.) Ball	Oukaïmeden, Morocco	31°12′11.8″N, 7°51′50.4″W	5	SEV 284708
*Sonchus canariensis* (Sch. Bip.) Boulos	Buenavista del Norte, Tenerife (TE), Spain	28°18′41.2″N, 16°50′52.0″W	5	SKK060305 SKK060306
*Sonchus palmensis* (Webb) Boulos	Breña Baja, La Palma (PA), Spain	28°38′06.4″N, 17°46′40.2″W	5	SKK060324

Note

*N* = number of individuals per population.

a Vouchers were deposited at the Ha Eun Herbarium (SKK; Sungkyunkwan University, Korea) and the herbarium of the Department of Plant Biology and Ecology (SEV; University of Seville, Spain).
